# Supplementary material for: Sustained software development, not number of citations or journal choice, is indicative of accurate bioinformatic software
Source: Genome Biol. 2022 Feb 16;23:56. doi: 10.1186/s13059-022-02625-x (PMC8851831; doi:10.1186/s13059-022-02625-x)
Supplement: Supplementary file 1 — Additional file 1 Supplementary Figures S1-S8 and the neutral software benchmark reference list the accuracy and speed data is derived from [1-66]. [file 13059_2022_2625_MOESM1_ESM.pdf]

Supplementary results for: Sustained software development, not number of citations or journal choice, is indicative of accurate bioinformatic software

Paul P. Gardner[1,2]*, James M. Paterson[3], Stephanie McGimpsey[4], Fatemeh Ashari-Ghomi[5], Sinan U. Umu[6], Aleksandra Pawlik[7], Alex Gavryushkin[8], Michael A Black[1]

Abstract
In the below we provide additional results for our investigation of computational biology benchmarks.

Keywords
computational biology — accuracy — benchmarks — meta-analysis — software development

[1] *Department of Biochemistry, University of Otago, Dunedin, New Zealand.*
[2] *Biomolecular Interaction Centre, University of Canterbury, Christchurch, New Zealand.*
[3] *Department of Civil and Natural Resources Engineering, University of Canterbury, Christchurch, New Zealand.*
[4] *Parasites and Microbes, Wellcome Sanger Institute, Wellcome Genome Campus, Hinxton, 8 Cambridgeshire, CB10 1RQ, UK.*
[5] *Research Group for Genomic Epidemiology, National Food Institute, Technical University of Denmark, Kongens Lyngby, Denmark.*
[6] *Department of Research, Cancer Registry of Norway, Oslo, Norway.*
[7] *New Zealand eScience Infrastructure, 49 Symonds St, Auckland, New Zealand.*
[8] *Department of Computer Science, University of Otago, Dunedin, New Zealand.*
***Corresponding author**: paul.gardner@otago.ac.nz

Literature mining

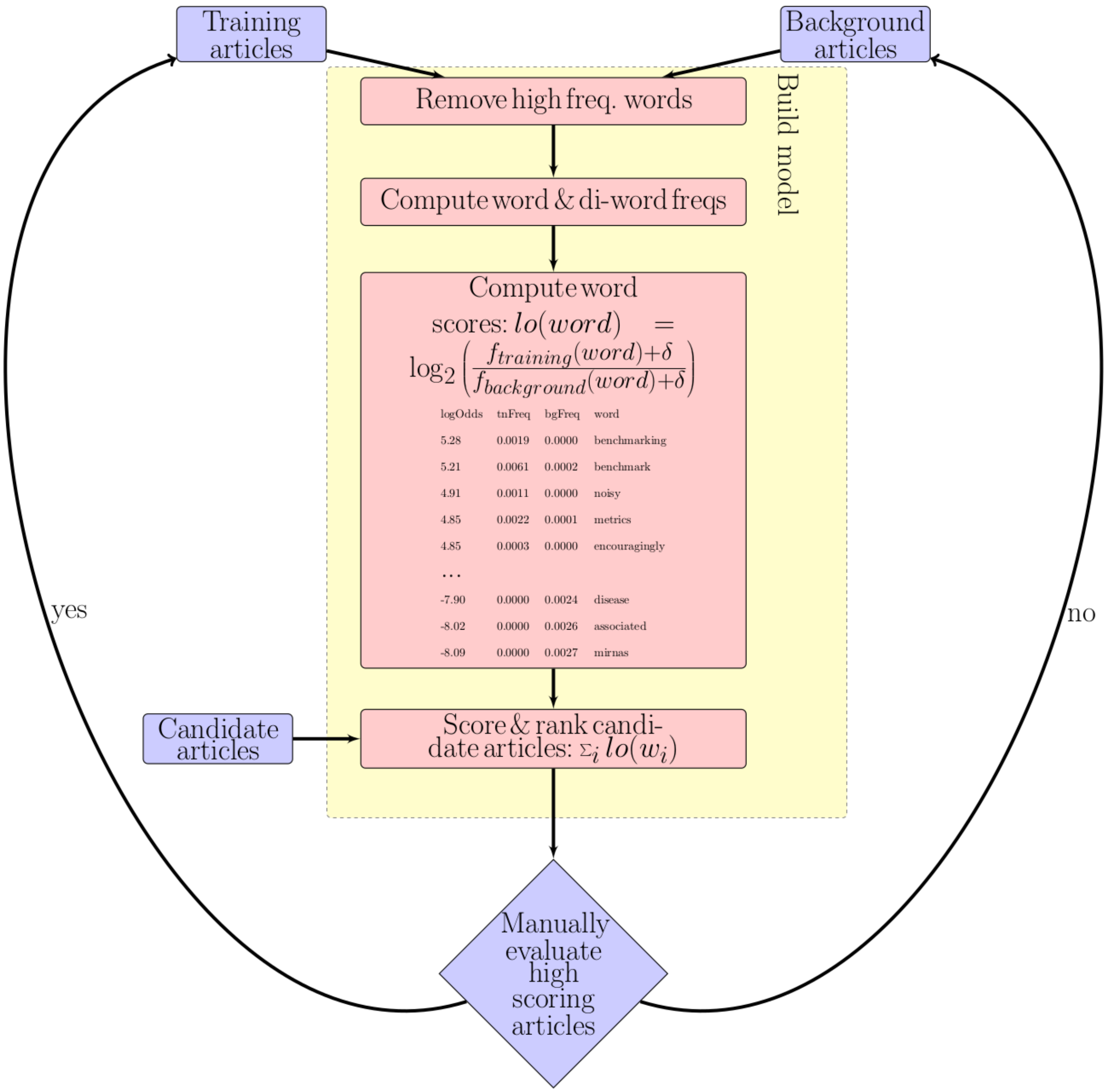

Figure S1. In order to improve the identification of benchmark articles that rank both accuracy and speed we developed a tool for ranking PubMed articles based upon word association scores (measured in 'bits'). In brief, keywords were extracted from titles and abstracts for both training (in this case benchmark articles) and background articles (articles published between 2013 and 2015 with "bioinformatics" in the title or abstract). Log-odds ratios were computed for each keyword (measured in 'bits'). Candidate articles that matched a hand-selected list of keywords associated with benchmarks: `((bioinformatics OR (computational AND biology)) AND (algorithmic OR algorithms OR biotechnologies OR computational OR kernel OR methods OR procedure OR programs OR software OR technologies)) AND (accuracy OR analysis OR assessment OR benchmark OR benchmarking OR biases OR comparing OR comparison OR comparisons OR comparative OR comprehensive OR effectiveness OR estimation OR evaluation OR metrics OR efficiency OR performance OR perspective OR quality OR rated OR robust OR strengths OR suitable OR suitability OR superior OR survey OR weaknesses OR correctness OR correct OR evaluate OR competing OR competition) AND (complexity OR cputime OR runtime OR walltime OR duration OR elapsed OR fast OR faster OR perform OR performance OR slow OR slower OR speed OR time OR (computational AND resources))`. These were then scored and ranked with a "sum of bits" score. High ranking articles were then inspected, those that met our criteria were added to the training set, those that did not were added to the background set of articles.

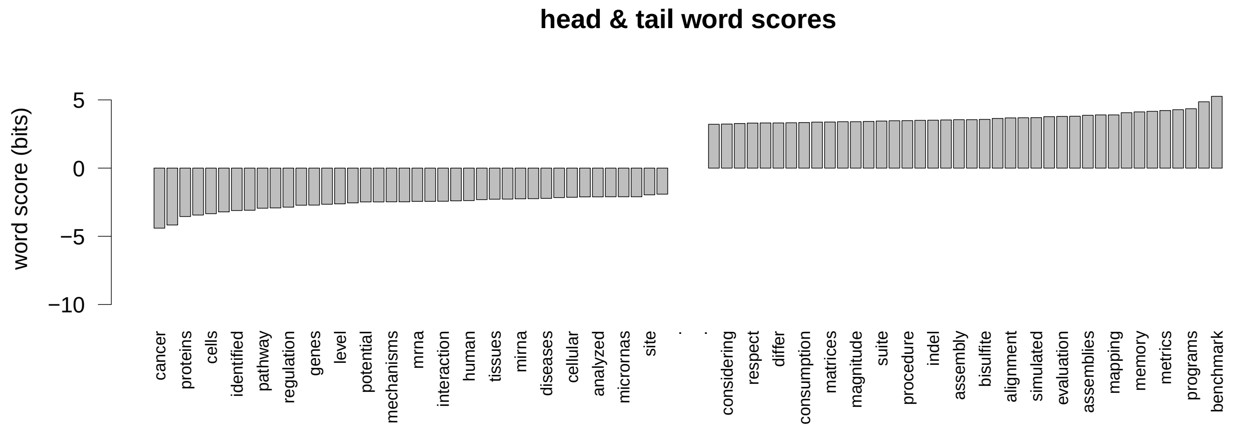

Figure S2. The 40 highest (**right**) and lowest (**left**) scoring words that are associated with bioinformatic benchmark articles from the training benchmark articles, compared to the background articles. The log-odds ratios (measured in bits) are indicated on the y-axis.

Data visualisations

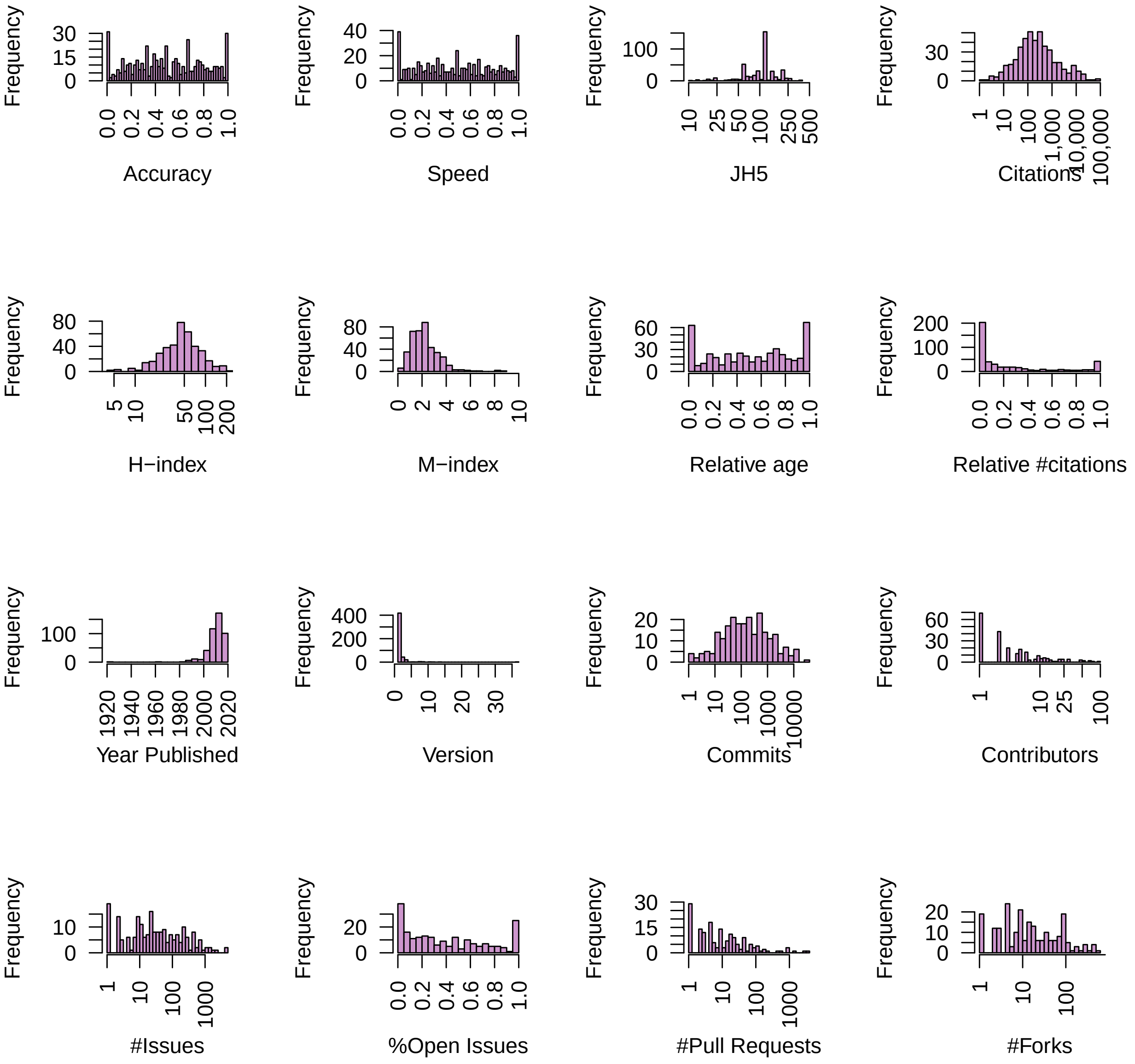

Figure S3. The distributions for the measures that have been proposed to be predictive of software quality used in this study. These are, reading from left to right, top to bottom: Accuracy – the mean normalised accuracy rank for each benchmarked method; Speed – the mean normalised speed rank for each benchmarked method; JH5 – the Journal H5 index to the highest impact journal that has published a manuscript describing a method, data from GoogleScholar 2020 Metrics; Cites – the number of citations to the most cited manuscript describing a method, data from GoogleScholar; H-index – the H-index for the highest profile corresponding author from the manuscripts describing a method, data from GoogleScholar User Profiles; M-index – the M-index (H-index/(#years since first publication)) for the highest profile corresponding author from the manuscripts describing a method, data from GoogleScholar User Profiles; Relative ages – for different tools, for each benchmark tools were ranked based upon publication dates, these ranks were normalised to lie between 0 and 1. Relative #citations – within each benchmark, what is the relative age of each tool, this corrects for older research fields (e.g. sequence alignment) vs newer research fields (e.g. single-cell sequencing); Year published – the first year each software tool was published; Version – the most resent version number for each tool; Commits – the number of commits to Github; Contributors – the number of contributors to a Github repository; #Issues – the number of open and closed issues for each Github repository; %Open issues – the pecentage of issues that remain open for each Github repository; #Pull requests – the number of pull requests to a Github repository; #Forks – the number of times a Github repository has been forked;

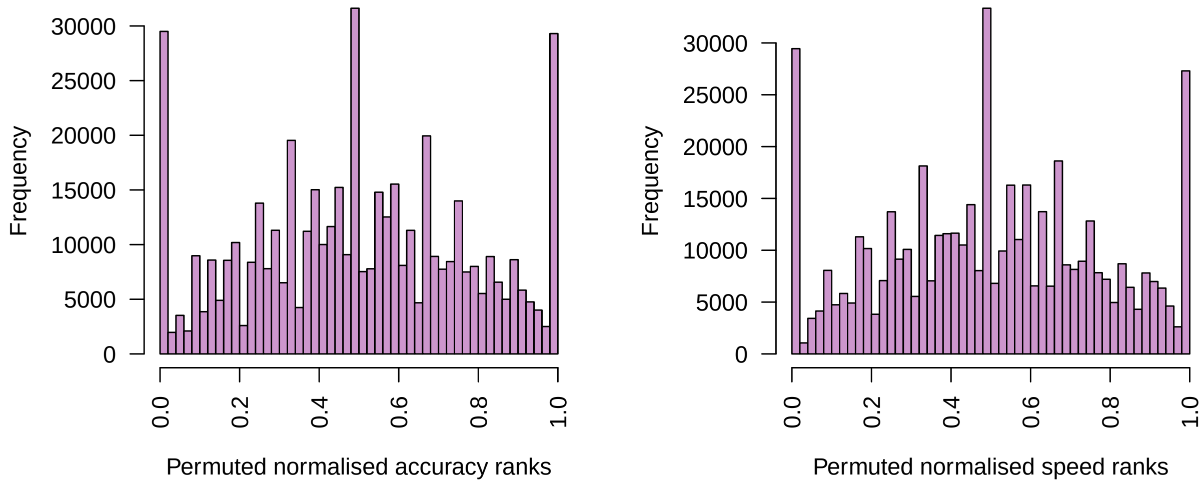

Figure S4. The distributions for the permuted accuracy (**left**) and speed (**right**) values, where accuracy and speed ranks have been normalised to lie between 0 and 1.

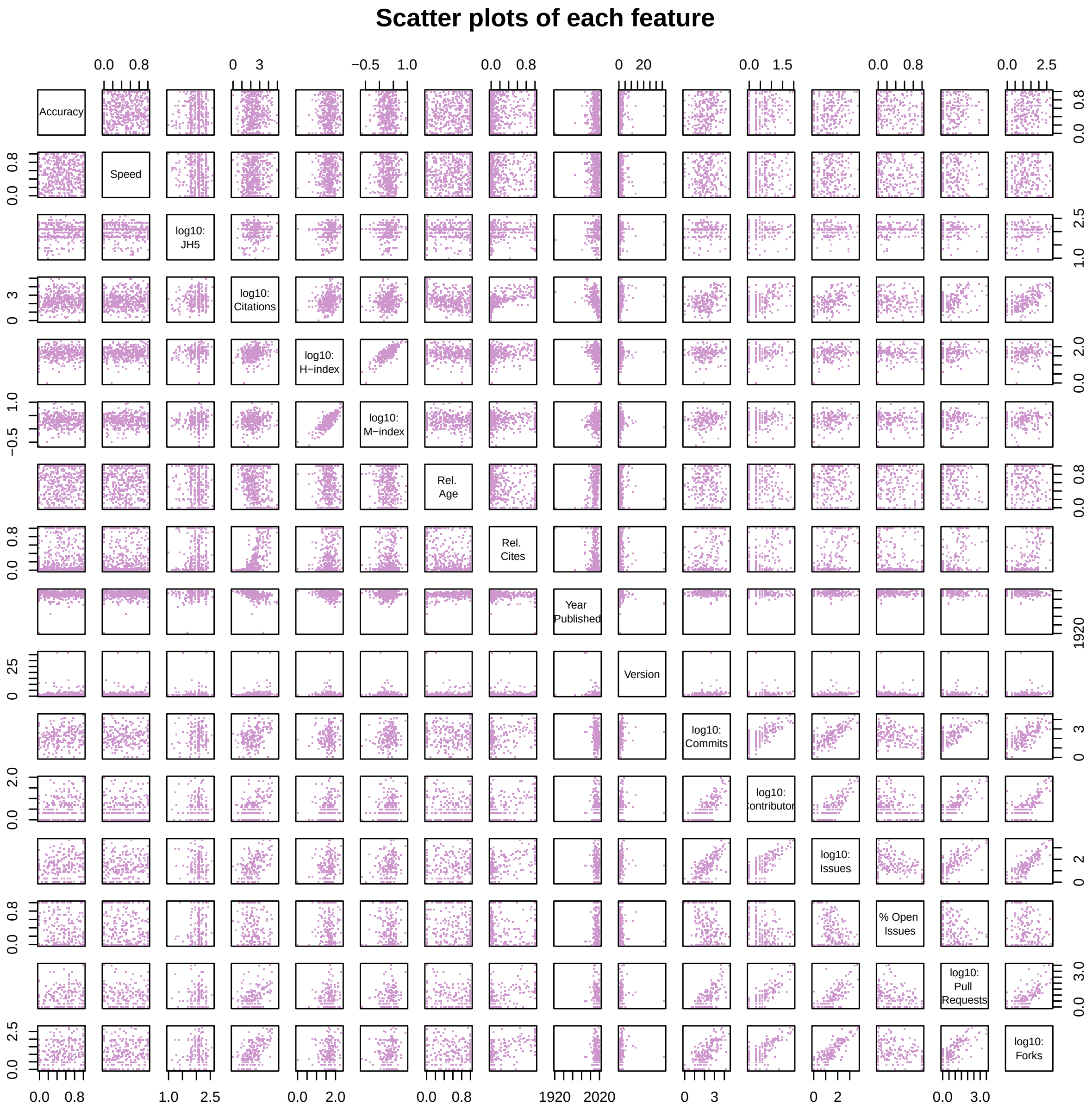

Figure S5. Dot plots for each feature relative to every other feature (accuracy, speed, JH5, citations, H-index, M-index, relAge, relCites, yearPublished, version, commits, contributors, #Issues, %Open issues, #Pull requests, and the #Forks).

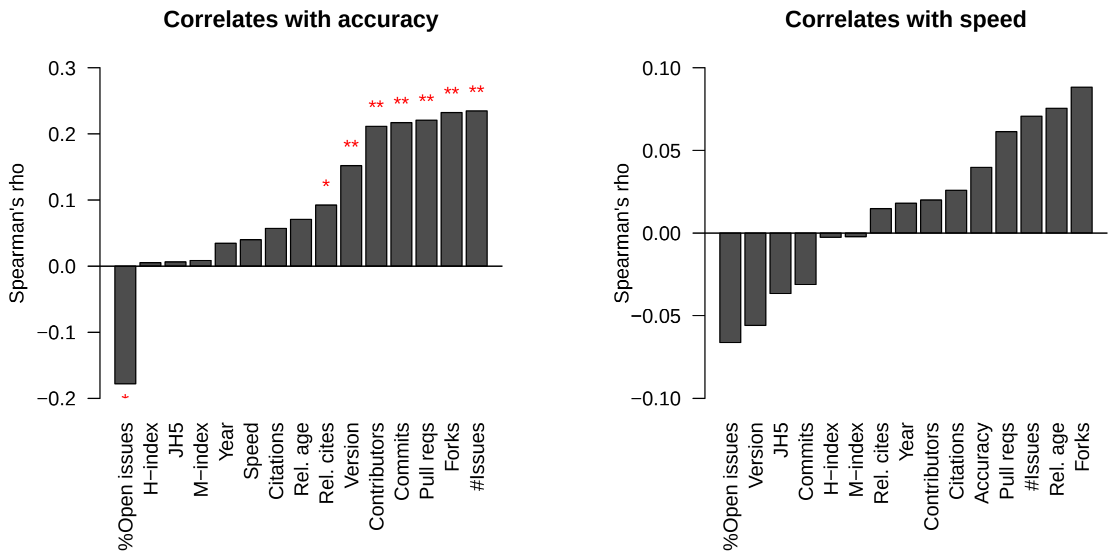

Figure S6. The correlation between method accuracy (**left**) and method speed (**right**) and measures we expected to be predictive of software quality. E.g. author reputation measures (H-index, M-index), journal reputation (JH5 and JIF), number of users (citations and relative citations) or the recency of methods (year and relative age). The correlations were estimated using Spearman's ρ. The signficant relationships are indicated with a "*".

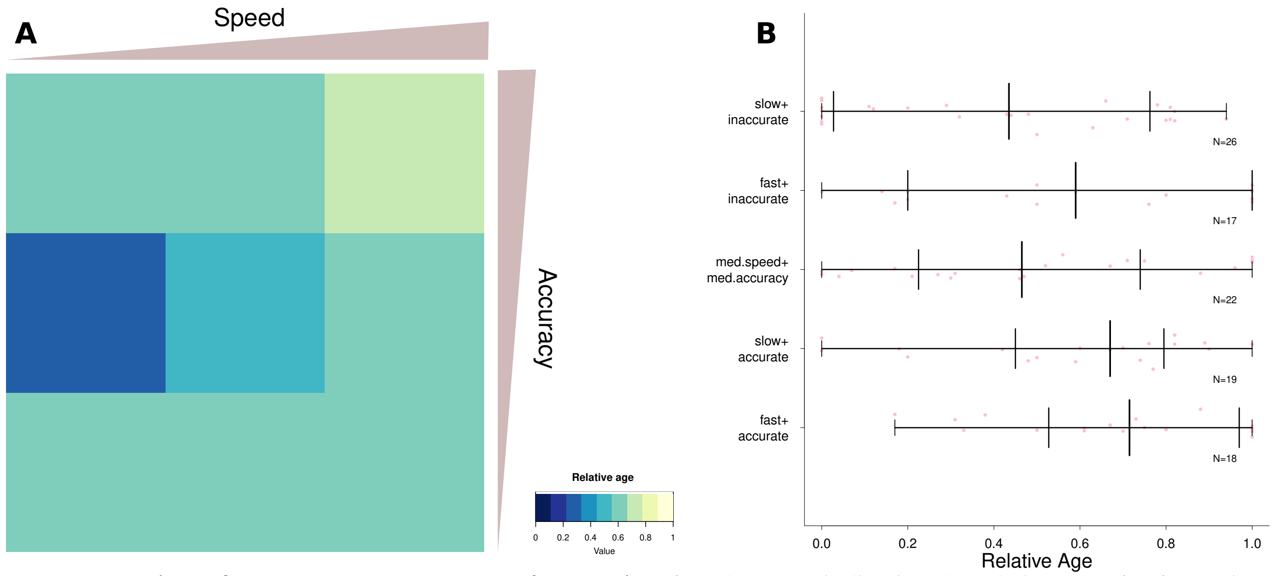

Figure S7. Relative software tool ages and speeds/accuracies. A. A heatmap indicating the relative age of software in a range of relative accuracy and speed rankings. Blue colours indicate an abundance of older software tools in an accuracy and speed category, while light colours indicate younger software in an accuracy and speed category. **B.** Jitter-plots for the relative age distribution for software tools in each of the 5 3x3 cells indicated in **B**. The five row correspond to the four extreme corners of the speed vs accuracy spectrum (i.e. slow and inaccurate, slow and accurate, fast and inaccurate, fast and accurate) and the central box (medium speed and medium accuracy). Median, quartile and extreme values are indicated with tick marks for each group. Accurate and fast software tools tend to have been published more recently than inaccurate and slow tools (Wilcox rank sum test, $W = 341,\ P = 5 \times 10^{-3}$)

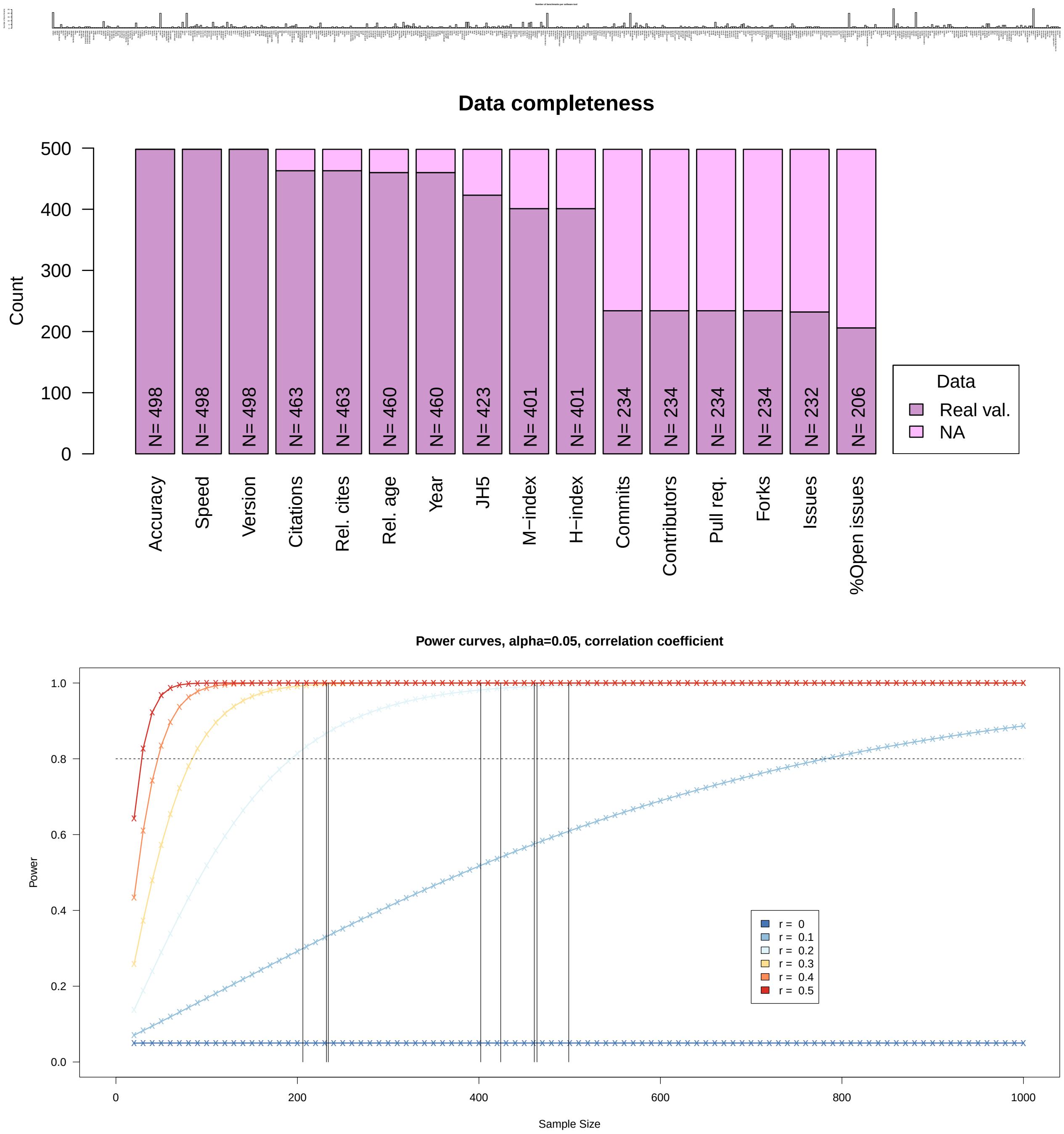

Figure S8. Top: The number of benchmarks each software tool has appeared in. **Middle:** Plot of the amount missing/known data for each of the software features used in this analysis. **Bottom:** a power curve analysis shows the sample sizes for each set of features is sufficient to detect correlations $r \geq 0.2$ between our features of interest with $power \geq 0.8$. For example, for speed vs accuracy $N = 498$, which is indicated with a vertical line.

The complete list of benchmarks used for this study [1, 2, 3, 4, 5, 6, 7, 8, 9, 10, 11, 12, 13, 14, 15, 16, 17, 18, 19, 20, 21, 22, 23, 24, 25, 26, 27, 28, 29, 30, 31, 32, 33, 34, 35, 36, 37, 38, 39, 40, 41, 42, 43, 44, 45, 46, 47, 48, 49, 50, 51, 52, 53, 54, 55, 56, 57, 58, 59, 60, 61, 62, 63, 64, 65, 66].

References

[1] K. Mitchell, J. J. Brito, I. Mandric, Q. Wu, S. Knyazev, S. Chang, L. S. Martin, A. Karlsberg, E. Gerasimov, R. Littman, B. L. Hill, N. C. Wu, H. T. Yang, K. Hsieh, L. Chen, E. Littman, T. Shabani, G. Enik, D. Yao, R. Sun, J. Schroeder, E. Eskin, A. Zelikovsky, P. Skums, M. Pop, and S. Mangul. Benchmarking of computational error-correction methods for next-generation sequencing data. *Genome Biol*, 21(1):71, 03 2020.

[2] L. Zhao, H. Liu, X. Yuan, K. Gao, and J. Duan. Comparative study of whole exome sequencing-based copy number variation detection tools. *BMC Bioinformatics*, 21(1):97, Mar 2020.

[3] H. T. N. Tran, K. S. Ang, M. Chevrier, X. Zhang, N. Y. S. Lee, M. Goh, and J. Chen. A benchmark of batch-effect correction methods for single-cell RNA sequencing data. *Genome Biol*, 21(1):12, 01 2020.

[4] F. Luo. A systematic evaluation of copy number alterations detection methods on real SNP array and deep sequencing data. *BMC Bioinformatics*, 20(Suppl 25):692, Dec 2019.

[5] R. R. Wick and K. E. Holt. Benchmarking of long-read assemblers for prokaryote whole genome sequencing. *F1000Res*, 8:2138, 2019.

[6] B. J. Haas, A. Dobin, B. Li, N. Stransky, N. Pochet, and A. Regev. Accuracy assessment of fusion transcript detection via read-mapping and de novo fusion transcript assembly-based methods. *Genome Biol*, 20(1):213, 10 2019.

[7] J. Bradford and D. Perrin. A benchmark of computational CRISPR-Cas9 guide design methods. *PLoS Comput Biol*, 15(8):e1007274, 08 2019.

[8] D. L. Cameron, L. Di Stefano, and A. T. Papenfuss. Comprehensive evaluation and characterisation of short read general-purpose structural variant calling software. *Nat Commun*, 10(1):3240, 07 2019.

[9] S. Kosugi, Y. Momozawa, X. Liu, C. Terao, M. Kubo, and Y. Kamatani. Comprehensive evaluation of structural variation detection algorithms for whole genome sequencing. *Genome Biol*, 20(1):117, 06 2019.

[10] L. Zhang, W. Bai, N. Yuan, and Z. Du. Comprehensively benchmarking applications for detecting copy number variation. *PLoS Comput Biol*, 15(5):e1007069, 05 2019.

[11] V. K. Raghu, A. Poon, and P. V. Benos. Evaluation of Causal Structure Learning Methods on Mixed Data Types. *Proc Mach Learn Res*, 92:48–65, Aug 2018.

[12] S. C. Manekar and S. R. Sathe. Estimating the k-mer Coverage Frequencies in Genomic Datasets: A Comparative Assessment of the State-of-the-art. *Curr Genomics*, 20(1):2–15, Jan 2019.

[13] W. Saelens, R. Cannoodt, H. Todorov, and Y. Saeys. A comparison of single-cell trajectory inference methods. *Nat Biotechnol*, 37(5):547–554, 05 2019.

[14] S. Fu, A. Wang, and K. F. Au. A comparative evaluation of hybrid error correction methods for error-prone long reads. *Genome Biol*, 20(1):26, 02 2019.

[15] T. Wang, B. Li, C. E. Nelson, and S. Nabavi. Comparative analysis of differential gene expression analysis tools for single-cell RNA sequencing data. *BMC Bioinformatics*, 20(1):40, Jan 2019.

[16] H. Lee, K. W. Lee, T. Lee, D. Park, J. Chung, C. Lee, W. Y. Park, and D. S. Son. Performance evaluation method for read mapping tool in clinical panel sequencing. *Genes Genomics*, 40(2):189–197, 2018.

[17] A. B. R. McIntyre, R. Ounit, E. Afshinnekoo, R. J. Prill, E. Hénaff, N. Alexander, S. S. Minot, D. Danko, J. Foox, S. Ahsanuddin, S. Tighe, N. A. Hasan, P. Subramanian, K. Moffat, S. Levy, S. Lonardi, N. Greenfield, R. R. Colwell, G. L. Rosen, and C. E. Mason. Comprehensive benchmarking and ensemble approaches for metagenomic classifiers. *Genome Biol*, 18(1):182, 09 2017.

[18] L. Song, W. Huang, J. Kang, Y. Huang, H. Ren, and K. Ding. Comparison of error correction algorithms for Ion Torrent PGM data: application to hepatitis B virus. *Sci Rep*, 7(1):8106, 08 2017.

[19] M. D. Ramstetter, T. D. Dyer, D. M. Lehman, J. E. Curran, R. Duggirala, J. Blangero, J. G. Mezey, and A. L. Williams. Benchmarking Relatedness Inference Methods with Genome-Wide Data from Thousands of Relatives. *Genetics*, 207(1):75–82, 09 2017.

[20] F. Zare, M. Dow, N. Monteleone, A. Hosny, and S. Nabavi. An evaluation of copy number variation detection tools for cancer using whole exome sequencing data. *BMC Bioinformatics*, 18(1):286, May 2017.

[21] L. Siegwald, H. Touzet, Y. Lemoine, D. Hot, C. Audebert, and S. Caboche. Assessment of Common and Emerging Bioinformatics Pipelines for Targeted Metagenomics. *PLoS One*, 12(1):e0169563, 2017.

[22] G. V. Saripella, E. L. Sonnhammer, and K. Forslund. Benchmarking the next generation of homology inference tools. *Bioinformatics*, 32(17):2636–2641, 09 2016.

[23] S. Kumar, A. D. Vo, F. Qin, and H. Li. Comparative assessment of methods for the fusion transcripts detection from RNA-Seq data. *Sci Rep*, 6:21597, Feb 2016.

[24] S. Lindgreen, K. L. Adair, and P. P. Gardner. An evaluation of the accuracy and speed of metagenome analysis tools. *Sci Rep*, 6:19233, Jan 2016.

[25] J. Tsuji and Z. Weng. Evaluation of preprocessing, mapping and postprocessing algorithms for analyzing whole genome bisulfite sequencing data. *Brief Bioinform*, 17(6):938–952, 11 2016.

[26] H. W. Huang, J. C. Mullikin, N. F. Hansen, B. B. Barnabas, R. W. Blakesley, G. G. Bouffard, S. Y. Brooks, H. Coleman, J. G. Dayal, L. Dekhtyar, M. Gregory, X. Guan, J. Han, S. L. Ho, R. Legaspi, Q. L. Maduro, C. A. Masiello, B. Maskeri, J. C. McDowell, C. Montemayor, J. C. Mullikin, M. Park, N. L. Riebow, K. Schandler, B. Schmidt, C. Sison, S. Stantripop, J. W. Thomas, P. J. Thomas, M. Vemulapalli, and A. C. Young. Evaluation of variant detection software for pooled next-generation sequence data. *BMC Bioinformatics*, 16:235, Jul 2015.

[27] O. J. Rackham, P. Dellaportas, E. Petretto, and L. Bottolo. WGBSSuite: simulating whole-genome bisulphite sequencing data and benchmarking differential DNA methylation analysis tools. *Bioinformatics*, 31(14):2371–2373, Jul 2015.

[28] A. Pain, A. Ott, H. Amine, T. Rochat, P. Bouloc, and D. Gautheret. An assessment of bacterial small RNA target prediction programs. *RNA Biol*, 12(5):509–513, 2015.

[29] M. T. Pervez, M. E. Babar, A. Nadeem, M. Aslam, A. R. Awan, N. Aslam, T. Hussain, N. Naveed, S. Qadri, U. Waheed, and M. Shoaib. Evaluating the accuracy and efficiency of multiple sequence alignment methods. *Evol Bioinform Online*, 10:205–217, 2014.

[30] M. M. Abbas, Q. M. Malluhi, and P. Balakrishnan. Assessment of de novo assemblers for draft genomes: a case study with fungal genomes. *BMC Genomics*, 15 Suppl 9:S10, 2014.

[31] R. Liu, A. E. Loraine, and J. A. Dickerson. Comparisons of computational methods for differential alternative splicing detection using RNA-seq in plant systems. *BMC Bioinformatics*, 15:364, Dec 2014.

[32] S. Jünemann, K. Prior, A. Albersmeier, S. Albaum, J. Kalinowski, A. Goesmann, J. Stoye, and D. Harmsen. GABenchToB: a genome assembly benchmark tuned on bacteria and benchtop sequencers. *PLoS One*, 9(9):e107014, 2014.

[33] H. Tran, J. Porter, M. A. Sun, H. Xie, and L. Zhang. Objective and comprehensive evaluation of bisulfite short read mapping tools. *Adv Bioinformatics*, 2014:472045, 2014.

[34] S. Caboche, C. Audebert, Y. Lemoine, and D. Hot. Comparison of mapping algorithms used in high-throughput sequencing: application to Ion Torrent data. *BMC Genomics*, 15:264, Apr 2014.

[35] F. S. Pais, P. C. Ruy, G. Oliveira, and R. S. Coimbra. Assessing the efficiency of multiple sequence alignment programs. *Algorithms Mol Biol*, 9(1):4, Mar 2014.

[36] M. Maška, V. Ulman, D. Svoboda, P. Matula, P. Matula, C. Ederra, A. Urbiola, T. España, S. Venkatesan, D. M. Balak, P. Karas, T. Bolcková, M. Streitová, C. Carthel, S. Coraluppi, N. Harder, K. Rohr, K. E. Magnusson, J. Jaldén, H. M. Blau, O. Dzyubachyk, P. Křížek, G. M. Hagen, D. Pastor-Escuredo, D. Jimenez-Carretero, M. J. Ledesma-Carbayo, A. Muñoz-Barrutia, E. Meijering, M. Kozubek, and C. Ortiz-de Solorzano. A benchmark for comparison of cell tracking algorithms. *Bioinformatics*, 30(11):1609–1617, Jun 2014.

[37] D. Kleftogiannis, P. Kalnis, and V. B. Bajic. Comparing memory-efficient genome assemblers on stand-alone and cloud infrastructures. *PLoS One*, 8(9):e75505, 2013.

[38] M. S. Bayzid and T. Warnow. Naive binning improves phylogenomic analyses. *Bioinformatics*, 29(18):2277–2284, Sep 2013.

[39] A. Hatem, D. Bozdağ, A. E. Toland, and Ü. V. Çatalyürek. Benchmarking short sequence mapping tools. *BMC Bioinformatics*, 14:184, Jun 2013.

[40] A. Abdullah, S. Deris, M. S. Mohamad, and S. Anwar. An improved swarm optimization for parameter estimation and biological model selection. *PLoS One*, 8(4):e61258, 2013.

[41] B. Lu, Z. Zeng, and T. Shi. Comparative study of de novo assembly and genome-guided assembly strategies for transcriptome reconstruction based on RNA-Seq. *Sci China Life Sci*, 56(2):143–155, Feb 2013.

[42] A. L. Bazinet and M. P. Cummings. A comparative evaluation of sequence classification programs. *BMC Bioinformatics*, 13:92, May 2012.

[43] S. Schbath, V. Martin, M. Zytnicki, J. Fayolle, V. Loux, and J. F. Gibrat. Mapping reads on a genomic sequence: an algorithmic overview and a practical comparative analysis. *J Comput Biol*, 19(6):796–813, Jun 2012.

[44] X. Yang, S. P. Chockalingam, and S. Aluru. A survey of error-correction methods for next-generation sequencing. *Brief Bioinform*, 14(1):56–66, Jan 2013.

[45] Y. Li, Z. Zhang, F. Liu, W. Vongsangnak, Q. Jing, and B. Shen. Performance comparison and evaluation of software tools for microRNA deep-sequencing data analysis. *Nucleic Acids Res*, 40(10):4298–4305, May 2012.

[46] J. Yang and T. Warnow. Fast and accurate methods for phylogenomic analyses. *BMC Bioinformatics*, 12 Suppl 9:S4, Oct 2011.

[47] J. Shang, J. Zhang, Y. Sun, D. Liu, D. Ye, and Y. Yin. Performance analysis of novel methods for detecting epistasis. *BMC Bioinformatics*, 12:475, Dec 2011.

[48] K. Liu, C. R. Linder, and T. Warnow. RAxML and FastTree: comparing two methods for large-scale maximum likelihood phylogeny estimation. *PLoS One*, 6(11):e27731, 2011.

[49] M. Ruffalo, T. LaFramboise, and M. Koyutürk. Comparative analysis of algorithms for next-generation sequencing read alignment. *Bioinformatics*, 27(20):2790–2796, Oct 2011.

[50] M. Holtgrewe, A. K. Emde, D. Weese, and K. Reinert. A novel and well-defined benchmarking method for second generation read mapping. *BMC Bioinformatics*, 12:210, May 2011.

[51] S. Bao, R. Jiang, W. Kwan, B. Wang, X. Ma, and Y. Q. Song. Evaluation of next-generation sequencing software in mapping and assembly. *J Hum Genet*, 56(6):406–414, Jun 2011.

[52] J. D. Thompson, B. Linard, O. Lecompte, and O. Poch. A comprehensive benchmark study of multiple sequence alignment methods: current challenges and future perspectives. *PLoS One*, 6(3):e18093, Mar 2011.

[53] W. Zhang, J. Chen, Y. Yang, Y. Tang, J. Shang, and B. Shen. A practical comparison of de novo genome assembly software tools for next-generation sequencing technologies. *PLoS One*, 6(3):e17915, Mar 2011.

[54] K. Liu, C. R. Linder, and T. Warnow. Multiple sequence alignment: a major challenge to large-scale phylogenetics. *PLoS Curr*, 2:RRN1198, Nov 2010.

[55] D. Tikk, P. Thomas, P. Palaga, J. Hakenberg, and U. Leser. A comprehensive benchmark of kernel methods to extract protein-protein interactions from literature. *PLoS Comput Biol*, 6:e1000837, Jul 2010.

[56] M. S. Swenson, F. Barbançon, T. Warnow, and C. R. Linder. A simulation study comparing supertree and combined analysis methods using SMIDGen. *Algorithms Mol Biol*, 5:8, Jan 2010.

[57] K. Liu, S. Nelesen, S. Raghavan, C. R. Linder, and T. Warnow. Barking up the wrong treelength: the impact of gap penalty on alignment and tree accuracy. *IEEE/ACM Trans Comput Biol Bioinform*, 6(1):7–21, 2009.

[58] C. Yang, Z. He, and W. Yu. Comparison of public peak detection algorithms for MALDI mass spectrometry data analysis. *BMC Bioinformatics*, 10:4, Jan 2009.

[59] M. Oscamou, D. McDonald, V. B. Yap, G. A. Huttley, M. E. Lladser, and R. Knight. Comparison of methods for estimating the nucleotide substitution matrix. *BMC Bioinformatics*, 9:511, Dec 2008.

[60] E. Lange, R. Tautenhahn, S. Neumann, and C. Gröpl. Critical assessment of alignment procedures for LC-MS proteomics and metabolomics measurements. *BMC Bioinformatics*, 9:375, Sep 2008.

[61] S. Saha, S. Bridges, Z. V. Magbanua, and D. G. Peterson. Empirical comparison of ab initio repeat finding programs. *Nucleic Acids Res*, 36(7):2284–2294, Apr 2008.

[62] E. K. Freyhult, J. P. Bollback, and P. P. Gardner. Exploring genomic dark matter: a critical assessment of the performance of homology search methods on noncoding RNA. *Genome Res*, 17(1):117–125, Jan 2007.

[63] P. A. Nuin, Z. Wang, and E. R. Tillier. The accuracy of several multiple sequence alignment programs for proteins. *BMC Bioinformatics*, 7:471, Oct 2006.

[64] B. Wallner and A. Elofsson. All are not equal: a benchmark of different homology modeling programs. *Protein Sci*, 14(5):1315–1327, May 2005.

[65] R. Kolodny, P. Koehl, and M. Levitt. Comprehensive evaluation of protein structure alignment methods: scoring by geometric measures. *J Mol Biol*, 346(4):1173–1188, Mar 2005.

[66] Siew Teng Ng, Chuii Khim Chong, Yee Wen Choon, Lian En Chai, Safaai Deris, Rosli Md Illias, Mohd Shahir Shamsir, and Mohd Saberi Mohamad. Estimating kinetic parameters for essential amino acid production in arabidopsis thaliana by using particle swarm optimization. *Jurnal Teknologi*, 64(1), 2013.
