# Supplementary material for: Sustained software development, not number of citations or journal choice, is indicative of accurate bioinformatic software
Source: Genome Biol. 2022 Feb 16;23:56. doi: 10.1186/s13059-022-02625-x (PMC8851831; doi:10.1186/s13059-022-02625-x)
Supplement: Supplementary file 3 — Additional file 3 Review history. [file 13059_2022_2625_MOESM3_ESM.docx]

**1^st^ round**

**Reviewer 1**

Note: The manuscript indicates that a supplementary materials document is available. However, I did not see any supplementary materials document. If this is available, I would like to review it, as this could impact my evaluation (as explained below).

The goal of this paper is to explore the relationship between the accuracy of a given bioinformatics software and whether it is used by practitioners. This is a valuable objective. The authors provide a study in which they explore citation counts, author reputation, journal impact, etc., to see whether any of these statistics (which reflect reputation) are predictive of accuracy, and find that they are not. The study also suggests that publication bias is a problem and leads to the continued publication of papers that describe software that may not be the best choices for biologists.

All this is very laudatory and of interest. But the paper as written does not provide adequate information to understand the study methodology (and this remained true even after looking at the github site). I suspect the authors were trying to avoid overloading the reviewer with what might be seen as unnecessary details, but the result was unsatisfying to this reviewer. Here are some specific questions that would have been useful, even if put into a supplementary materials document. (I note that the study says they examined 499 bioinformatics tools from 69 benchmarks published between 2005 and 2020, but this is not enough information.)

* Which specific publications were used to perform the study (i.e., publications that remained after all the filtering was performed)?

* Which bioinformatics software were evaluated in this study? Which major software was not included, and why?

* Which benchmarks were allowed? Which common benchmarks were not included, and why?

In addition, my reading of the protocol used in this study led me to wonder about some choices. For example:

* The authors seem to have limited the benchmarks to only those that passed some purity test (described here as meeting the Boulesteix criteria). If I understand this correctly, this would omit any benchmark that was introduced in the same paper as a method and then used to evaluate the method. This seems an unreasonably harsh selection criterion for benchmarks. It might justify not including studies of methods designed by the authors of the benchmark, but why would it omit studies using that benchmark to evaluate other methods? Perhaps I misunderstood how they applied the Boulesteix criteria, but if I am not wrong, then this is too rigid.

* It looks like the extraction of "rank" for accuracy and speed was done through a manual read of the selected studies. If so, this should be stated clearly, and some documentation provided for how each publication was interpreted. See the next comment for concerns about this.

* Using "rank" to characterize speed and accuracy seems inadequate. Many papers that study methods rank the methods but the differences in performance may be so small as to be unimportant: for example, there may only be a difference of a few seconds in running time among the first 5 methods, so that rank is not important. Similarly, differences in accuracy between the top methods can be miniscule. Furthermore, "accuracy" itself depends on how methods are evaluated -- is precision or recall (or some other metric) used? Note that the relative performance of two methods can easily depend on the choice of criterion and also model condition. Furthermore, in a single study, it is often the case that the relative performance of two methods will depend on the specific datasets and question that is explored. How is this taken into account in this study?

All of this may not matter if the study is large enough that these issues can be overcome by volume of data, but it's not clear to me that this study is large enough to not worry about these issues. At a minimum, a detailed supplementary materials document would be needed to understand the study and evaluate its findings.

I find myself in a frustrating position of very much agreeing that the objectives of the study are good ones and I suspect the conclusions are probably correct, so that I'd like to see this study published. But the current version of the study does not provide enough information to evaluate it, and so I am uncomfortable with recommending publication. (Note that I did not have access to the supplementary materials, and so some of these issues may be dealt with by examining these.)

I also noted some less important writing issues, as indicated below (these are just a sample). The authors should carefully read the manuscript for typos, punctuation issues, etc.

* Page 1, left column, line 58. "efficient, instead" -> "efficient; instead"

* Page 2, left column, line 8. "studies, these" -> "studies; these"

* Page 4, left column, lines 47-48. The statement "high profile journals are run by editors and reviewers who carefully select and curate the best manuscripts". This is in my experience not reliably true. It's what we hope is true, but is not necessarily true. Perhaps rewrite to say "Many believe that…"

* Page 4, right column, top line. See "level of hardware optimization [45,46]; However". Perhaps change "However" to "however"

* Page 5, left column, line 53. "Supplementary Figure ????"

* Page 6, right column, line 34. "never-the-less" -> "nevertheless"

* Inconsistent presentation of bibliography items (sometimes first names given, sometimes only first initials).

**Reviewer 2**

The authors describe an analysis of scientific software in which they compare accuracy and speed with characteristics of the authors, publications, and underlying software. This is an important paper. There are quite a few claims in the article that could really use a bit of additional digging. Finally, given the work done, there are a couple small analyses that I think would supplement the work because they would be unlikely to stand on their own as their own publication but that would add to the literature.

My concerns are:

* Figure 2A has very little information per pixel. Would it be possible to plot each software package (potentially sized by the number of studies on which a software tool was included)?

* Presumably the data are not fully complete (i.e., some scholar profiles couldn't be identified, etc). A table within either the methods or results breaking out the completeness of the records would be helpful.

* The number of different commits is one metric. However, different models for software development and/or squash-merge behaviors implemented by GitHub can make commits different to compare across projects. If the authors could also add the number of pull requests as well as a measure of the extent to which the code has changed since the initial commits (i.e., from 10+ commits onward or similar). Either of these might be a more accurate measure of the extent to which code is revised. One would hypothesize, based on the author's results, that this would be the most effective software but it would be interesting to see if the analysis bears this out.

* It would be great to add a count of GitHub issues (either total, closed, or both) to the statistics examined.

* Certain supplementary references appear to be built incorrectly (????).

**Reviewer 3**

In this manuscript, Gardner et al. conduct a large-scale empirical study of the correlates of "accuracy" of bioinformatics software. Looking across a wide-range of different metrics they find that most common metrics are, in fact, uncorrelated with the accuracy of the resulting software. For example, there is little to no correlation between the citations to an article and the method's accuracy or the h (or m) indices of the corresponding author. This result actually doesn't surprise me. They go on to demonstrate that, in fact, the only metric that is statistically significantly correlated with their adopted definition of accuracy (which is reasonable) is the number of commits to the repository/source code of the tool. There are also weak (but not statistically significant) correlations with the version of the tool and the number of contributors. Again, this result, while certainly important to quantify, seems intuitive --- software that is maintained and continuously developed has the opportunity to benefit from updates to its core algorithms, from bug fixes, and from feedback from users, unlike software that is abandoned. I have no major "concerns" with the manuscript per se, though I describe a bit below what I think would improve the manuscript:

* I think the article may benefit (and I would like to see) a bit more in the way of plausible interpretations of the results. For example, the authors show a scarcity of methods that perform "in the middle" in terms of speed and accuracy. They posit a plausible mechanism for this, namely that there may be a publication-bias against methods that honestly present themselves as making compromises rather than as being the "fastest" or the "most accurate" (claims that themselves may be dubious). Though it is, of course, difficult to gather evidence in support of this specific mechanism, I think this is important to highlight, because it suggests a way in which the community may be shortchanging itself, and points out a manner in which the editorial policies of certain journals may be doing a disservice to the field. Do the authors have putative mechanism that may describe the the lack of correlation between citations and accuracy? It is suggested that citations are likely more a reflection of "user-friendliness or the Matthew Effect other than accuracy", which may well be true. One other mechanism I might suggest is the following. The first methods published to solve a problem (or the first to adopt a fundamentally new approach) are more likely to receive a warm reception in "high impact" venues (leading to increased citations). Yet, without consistent development effort and improvements, the best versions of these ideas are likely to be implemented in follow-up work which, for the same reasons of publication-bias as already explained, might find good homes but are unlikely to make their way into the same journals. Such mechanisms, if true, would suggest that the apparent lack of correlation between "impact-based metrics" and accuracy may be, in part, a self-inflicted shortcoming resulting from the very novelty or "impact" bias propagated among certain publishers. How might the community start to address such issues or improve this situation?

* The continued development of a method, as measured by commits, is correlated with accuracy. Interestingly, commits are also statistically significantly correlated with the citations and relative citations (and less surprisingly, with version and number of contributors). What does the correlation between commits and citations imply? Given the correlation between commits and accuracy, how might we interpret the lack of the transitive correlation between citations and accuracy?

* Finally, the authors propose that the community might take steps to reduce the publication-bias away from "in the middle" tools to reduce their underrepresentation in the field given their potential utility. Likewise, what might the community do to foster better sustained development of tools? Given the importance of sustained development in providing accurate tools, how might the community reward and encourage such a practice? Given the prevalence of such tools being developed in academia, and the general lack of incentives to spend time on software maintenance and sustained development compared to other activities, how might the academic community further incentivize this, presumably desirable activity.

Minor:

* I noticed some missing references (e.g. line 53 of page 5, there is a reference to supplementary figure ????).

**Authors’s response**

Reviewer #1: Note: The manuscript indicates that a supplementary materials document is available. However, I did not see any supplementary materials document. If this is available, I would like to review it, as this could impact my evaluation (as explained below).

**● I am not sure what has happened to the supplementary materials, either we neglected to upload it, or Genome Biology doesn’t support these. Nevertheless, the supplementary materials are available on bioRxiv (https://www.biorxiv.org/content/10.1101/092205v3) and GitHub (in the manuscript and data directories) -- this should have been available through the journal's editorial management system.**

**● In addition, we have added an excel document of our curated datasets to the GitHub repository for those users who prefer to view these, rather than the raw, tab-delimited text files we use.**

The goal of this paper is to explore the relationship between the accuracy of a given bioinformatics software and whether it is used by practitioners. This is a valuable objective. The authors provide a study in which they explore citation counts, author reputation, journal impact, etc., to see whether any of these statistics (which reflect reputation) are predictive of accuracy, and find that they are not. The study also suggests that publication bias is a problem and leads to the continued publication of papers that describe software that may not be the best choices for biologists.

All this is very laudatory and of interest. But the paper as written does not provide adequate information to understand the study methodology (and this remained true even after looking at the github site). I suspect the authors were trying to avoid overloading the reviewer with what might be seen as unnecessary details, but the result was unsatisfying to this reviewer. Here are some specific questions that would have been useful, even if put into a supplementary materials document. (I note that the study says they examined 499 bioinformatics tools from 69 benchmarks published between 2005 and 2020, but this is not enough information.)

● **These were freely available in the**

**“data/speed-vs-accuracy-toolRanks2005-2020.tsv” and**

**“data/speed-vs-accuracy-toolInfo2005-2020.tsv” files in the github repository. Now an excel sheet has been added too. I have also extended the README to better describe file contents.**

* Which specific publications were used to perform the study (i.e., publications that remained after all the filtering was performed)?

**● Freely available in Supplementary Table 1 (and**

**speed-vs-accuracy-toolRanks2005-2020.tsv on GitHub).**

* Which bioinformatics software were evaluated in this study? Which major software was not included, and why?

**● See above**

* Which benchmarks were allowed? Which common benchmarks were not included, and why?

**● As mentioned in the manuscript, benchmarks were excluded if they introduced a new method, the authors had been involved in the development of a benchmarked tool, other reasons for exclusion included: too few tools, or in some cases inaccessible results (unable to parse ranks from figures, tables or supplementary materials. As can be seen in the “data/checked-pmids.tsv” file, more than 400 manuscripts were manually checked against our inclusion criteria. We have updated the “Criteria for inclusion” subsection to clarify these.**

In addition, my reading of the protocol used in this study led me to wonder about some choices. For example:

* The authors seem to have limited the benchmarks to only those that passed some purity test (described here as meeting the Boulesteix criteria). If I understand this correctly, this would omit any benchmark that was introduced in the same paper as a method and then used to evaluate the method. This seems an unreasonably harsh selection criterion for benchmarks. It might justify not including studies of methods designed by the authors of the benchmark, but why would it omit studies using that benchmark to evaluate other methods? Perhaps I misunderstood how they applied the Boulesteix criteria, but if I am not wrong, then this is too rigid.

**● The reviewer is correct, we deliberately exclude assessments of software by the same people who develop a tool. As Boulesteix describes in her papers, these assessments by "expert users" often do not reflect the real-world accuracy of applications in the hands of naive users. The severity of this problem in a particular field was recently quantified in a recent manuscript (https://genomebiology.biomedcentral.com/articles/10.1186/s13059-021-02365-4 ) -- in summary we found that 94.7% of non-neutral papers rank a newly introduced tool higher than competing methods, whereas for neutral comparisons these same new methods outperformed just 64.3% of their paired competitors. This highlights a marked bias in non-neutral benchmarks that we wish to avoid here.**

**● We have edited the MS to try to clarify the reason for this.**

* It looks like the extraction of "rank" for accuracy and speed was done through a manual read of the selected studies. If so, this should be stated clearly, and some documentation provided for how each publication was interpreted. See the next comment for concerns about this.

**● Until published datasets are provided in consistent, machine-readable formats, the means to extract ranks are necessarily heterogeneous. The authors acquired ranks from raw data whenever these were made available, in other instances these were manually extracted from manuscript figures and tables.**

**● The source of each set of ranks is given in Supplementary Table 1. ● We have updated the methods description to better describe the data acquisition process.**

* Using "rank" to characterize speed and accuracy seems inadequate. Many papers that study methods rank the methods but the differences in performance may be so small as to be unimportant: for example, there may only be a difference of a few seconds in running time among the first 5 methods, so that rank is not important. Similarly, differences in accuracy between the top methods can be miniscule. Furthermore, "accuracy" itself depends on how methods are evaluated -- is precision or recall (or some other metric) used?

Note that the relative performance of two methods can easily depend on the choice of criterion and also model condition. Furthermore, in a single study, it is often the case that the relative performance of two methods will depend on the specific datasets and question that is explored. How is this taken into account in this study?

**● Indeed, as the reviewer notes, there are many alternative measures of "accuracy", we have outlined in detail many of the more widely used measures across bioinformatics (https://doi.org/10.1186/s13059-019-1738-8). This has meant that it was impractical to restrict our study to benchmarks that use a single statistic such as the F1-score, or to compute this ourselves from generally incomplete, unavailable or inappropriate data. In addition these metrics can be computed quite differently by different groups, depending on how values for TP, FP, etc are defined. Hence for this study we have used the relative accuracy and speed ranks which is a pragmatic solution to our problem from which meaningful results can be gleaned. As Reviewer #3 notes, this is a reasonable solution to the problem.**

**● We make the assumption that tools that are highly ranked on one accuracy metric are generally also highly ranked on other metrics. Likewise, we assume that in general tools that rank highly on one dataset will rank highly on others. We acknowledge this won't always be the case, but one of the signs of a good benchmark is that they are robust (Weber *et al*. (2019)).**

**● Non-parametric, rank based statistics certainly have their place, see the literature around Spearman’s rho and Kendall’s tau for example.**

**● We have updated the methods description to better describe this.** All of this may not matter if the study is large enough that these issues can be overcome by volume of data, but it's not clear to me that this study is large enough to not worry about these issues. At a minimum, a detailed supplementary materials document would be needed to understand the study and evaluate its findings.

**● As described in the manuscript we have gathered results for 499 tools, from 134 benchmarks published in 69 manuscripts.**

**● As the below power curves illustrate, our sample size (indicated with a vertical line) is sufficient to detect all but the weakest correlations between our features of interest. This has been added to Figure S8.**

**● Based upon this, we see this is a more than sufficient set to support the primary claims of our paper. Especially given how labour intensive collection is, and relatively few neutral benchmarks there are**

I find myself in a frustrating position of very much agreeing that the objectives of the study are good ones and I suspect the conclusions are probably correct, so that I'd like to see this study published. But the current version of the study does not provide enough information to evaluate it, and so I am uncomfortable with recommending publication. (Note that I did not have access to the supplementary materials, and so some of these issues may be dealt with by examining these.)

**● Hopefully you can locate at least one copy of the Supplementary Materials for the revised version. These are available in the GitHub repository, bioRxiv, and should be available from Genome Biology.**

I also noted some less important writing issues, as indicated below (these are just a sample). The authors should carefully read the manuscript for typos, punctuation issues, etc. * Page 1, left column, line 58. "efficient, instead" -> "efficient; instead" * Page 2, left column, line 8. "studies, these" -> "studies; these"

* Page 4, left column, lines 47-48. The statement "high profile journals are run by editors and reviewers who carefully select and curate the best manuscripts". This is in my experience not reliably true. It's what we hope is true, but is not necessarily true. Perhaps rewrite to say "Many believe that…"

* Page 4, right column, top line. See "level of hardware optimization [45,46]; However". Perhaps change "However" to "however"

* Page 5, left column, line 53. "Supplementary Figure ????"

* Page 6, right column, line 34. "never-the-less" -> "nevertheless"

* Inconsistent presentation of bibliography items (sometimes first names given, sometimes only first initials).

**● Thank you for bringing these to our attention, these have been amended now.**

Reviewer #2: The authors describe an analysis of scientific software in which they compare accuracy and speed with characteristics of the authors, publications, and underlying software. This is an important paper. There are quite a few claims in the article that could really use a bit of additional digging. Finally, given the work done, there are a couple small

analyses that I think would supplement the work because they would be unlikely to stand on their own as their own publication but that would add to the literature.

My concerns are:

* Figure 2A has very little information per pixel. Would it be possible to plot each software package (potentially sized by the number of studies on which a software tool was included)? **● We have generated the requested figure and found it very difficult to read. We’ve included it in the supplementary information (Figure S8).**

**● Previous iterations of Figure 2 had far more information per pixel (e.g. a 10x10 grid and embedded histograms). However we felt this was a distraction from the primary point we wish to make with this figure, so have elected to reduce it to a 3x3 grid and violin plots.**

* Presumably the data are not fully complete (i.e., some scholar profiles couldn't be identified, etc). A table within either the methods or results breaking out the completeness of the records would be helpful.

**● Yes, good point. We should have clearly documented this. A new figure (S8) has been added to the supplement for this. The raw data is available in Supplementary Table 2.**

* The number of different commits is one metric. However, different models for software development and/or squash-merge behaviors implemented by GitHub can make commits different to compare across projects. If the authors could also add the number of pull requests as well as a measure of the extent to which the code has changed since the initial commits (i.e., from 10+ commits onward or similar). Either of these might be a more accurate measure of the extent to which code is revised. One would hypothesize, based on the author's results, that this would be the most effective software but it would be interesting to see if the analysis bears this out.

**● Almost any meaningful metric derived from GitHub use will be susceptible to the issues of different software development behaviours the Reviewer mentions. We have looked at the proposed “git-diff (10 commits version) HEAD” and didn’t see an immediately practical solution to computing a metric that measures the amount of changes between repository versions. If the reviewer has any practical code that solves this, we would be interested in trying it.**

**● Furthermore, there is a major issue discriminating between large data files, figures or binaries vs executable code. Given the often poor adherence to file-name conventions it would be a fairly major curational task to distinguish these. Also, are the number of lines of code changes really likely to be a useful metric? Should comments, blank spaces, dependencies, etc. be distinguished from actual executable lines? This seems like an issue that could form part of a followup study.**

* It would be great to add a count of GitHub issues (either total, closed, or both) to the statistics examined.

**● Thanks for this suggestion. We have added the number of issues, % open issues, number of forks and number of pull requests as additional features. As the Reviewer could predict, these GitHub derived metrics are all strongly correlated with each other (see updated Figure 1). This has caused some changes in our results. With the extra features, due to multiple-testing**

**corrections, some formerly significant correlations are now no-longer significant, and some new significant correlations have emerged.**

* Certain supplementary references appear to be built incorrectly (????). **● These have been corrected in the updated manuscript. Thanks!**

Reviewer #3: In this manuscript, Gardner et al. conduct a large-scale empirical study of the correlates of "accuracy" of bioinformatics software. Looking across a wide-range of different metrics they find that most common metrics are, in fact, uncorrelated with the accuracy of the resulting software. For example, there is little to no correlation between the citations to an article and the method's accuracy or the h (or m) indices of the corresponding author. This result actually doesn't surprise me. They go on to demonstrate that, in fact, the only metric that is statistically significantly correlated with their adopted definition of accuracy (which is reasonable) is the number of commits to the repository/source code of the tool. There are also weak (but not statistically significant) correlations with the version of the tool and the number of contributors. Again, this result, while certainly important to quantify, seems intuitive --- software that is maintained and continuously developed has the opportunity to benefit from updates to its core algorithms, from bug fixes, and from feedback from users, unlike software that is abandoned. I have no major "concerns" with the manuscript per se, though I describe a bit below what I think would improve the manuscript:

**● This has been fairly common feedback from the experienced members of the bioinformatics community. Our own observations of common patterns across many benchmarks is what originally motivated our study. Nevertheless, we think it can be useful to have data that shows the importance of long-term software maintenance.**

* I think the article may benefit (and I would like to see) a bit more in the way of plausible interpretations of the results. For example, the authors show a scarcity of methods that perform "in the middle" in terms of speed and accuracy. They posit a plausible mechanism for this, namely that there may be a publication-bias against methods that honestly present themselves as making compromises rather than as being the "fastest" or the "most accurate" (claims that themselves may be dubious). Though it is, of course, difficult to gather evidence in support of this specific mechanism, I think this is important to highlight, because it suggests a way in which the community may be shortchanging itself, and points out a manner in which the editorial policies of certain journals may be doing a disservice to the field.

**● We had thought we were already dangerously close to over-interpreting the results with our speculation that publication bias could drive the disparities we have observed.**

**● We have made multiple attempts to simulate scenarios that could cause a dissociation between speed and accuracy -- particularly publication bias. However we determined after much effort that the parameter space for this experiment is huge, and the results are very sensitive to initial conditions.**

Do the authors have putative mechanism that may describe the the lack of correlation between citations and accuracy? It is suggested that citations are likely more a reflection of "user-friendliness or the Matthew Effect other than accuracy", which may well be true. One other mechanism I might suggest is the following. The first methods published to solve a problem (or the first to adopt a fundamentally new approach) are more likely to receive a warm reception in "high impact" venues (leading to increased citations). Yet, without consistent development effort and improvements, the best versions of these ideas are likely to be implemented in follow-up work which, for the same reasons of publication-bias as already explained, might find good homes but are unlikely to make their way into the same journals. Such mechanisms, if true, would suggest that the apparent lack of correlation between "impact-based metrics" and accuracy may be, in part, a self-inflicted shortcoming resulting from the very novelty or "impact" bias propagated among certain publishers. How might the community start to address such issues or improve this situation?

**● We don’t see a straightforward way to model the mechanisms that may result in low correlations between accuracy and citations, yet significant correlations between GitHub activity and both accuracy and citations.**

**● We have clarified some areas of the conclusion that hopefully reflect the Reviewers excellent suggestions.**

**● We also don’t see too many straightforward ways to address the situation. We suggest less focus on novelty, and more on research rigor and reproducibility which may be one way forward.**

* The continued development of a method, as measured by commits, is correlated with accuracy. Interestingly, commits are also statistically significantly correlated with the citations and relative citations (and less surprisingly, with version and number of contributors). What does the correlation between commits and citations imply? Given the correlation between commits and accuracy, how might we interpret the lack of the transitive correlation between citations and accuracy?

**● Our first thought is that this may be due to leverage -- i.e. a few tools with high numbers of edits may also be highly cited and these could inflate the correlation. Although our use of Spearman’s correlation should somewhat mitigate this. A leverage analysis showed that this is unlikely to be the case.**

**● Our second thought is that “year” or “age” may be a major confounder as both measures increase monotonically over time. However there appears to be little association between age and number of commits (presumably because GitHub is a relatively recent resource).**

**● It is possible that the number of commits and citations are genuinely linked to each other. High-usage may encourage further development. Conversely, continuously updated software may be more likely to be used. On the other hand, it could also be a spurious correlation.**

* Finally, the authors propose that the community might take steps to reduce the publication-bias away from "in the middle" tools to reduce their underrepresentation in the field given their potential utility. Likewise, what might the community do to foster better sustained development of tools? Given the importance of sustained development in providing accurate tools, how might the community reward and encourage such a practice? Given the prevalence of such tools being developed in academia, and the general lack of incentives to spend time on software maintenance and sustained development compared to other activities, how might the academic community further incentivize this, presumably desirable activity.

**● A recent piece by Adam Siepel goes into some detail about this issue: https://doi.org/10.1186/s13059-019-1763-7, we referenced it in the previous version of the manuscript. However, we have now added an additional paragraph to the conclusion that largely echoes the sentiments expressed by Professor Siepel.**

**● When we read Reviewer #3’s report we suggested the editor invite them to write an opinion piece to appear alongside our article. We’re not sure if this was acted upon or is a desired outcome for all parties.**

Minor:

* I noticed some missing references (e.g. line 53 of page 5, there is a reference to supplementary figure ????).

**● Thank you. These should now be corrected.**

**2^nd^ round**

**Reviewer 1**

In my earlier review, I asked specific questions that mainly had to do with understanding the research protocol -- how the publications were screened, why the papers that were rejected were rejected, which publications were included, what criteria were used to select the "best" methods, etc. To some extent these are answered and the manuscript is now in better shape. The low-level writing errors have been addressed. On the other hand, I still have concerns the study design and how it is described in the paper and in the files provided in their github repository. These are not

In particular, the authors point out a specific file (speed-vs-accuracytoolRanks2005-2020.tsv, available in their github repository) which provides some insight into how they are interpreting each paper. These interpretations are interesting and show substantial effort on the part of the people doing the annotation - but they also reveal some problems.

1. The way the authors characterize the Boulesteix criteria for inclusion, then at least one of the 69 publications that passed the acid test probably should not have passed (although perhaps the authors should revise what their requirements are). Specifically, Thompson et al., "A comprehensive benchmark study of multiple sequence alignments: Current Challenges and Future Perspectives", is in the list, and this paper compares ClustalW to many other alignment methods. But Julie Thompson is also the first author on the paper for ClustalW. So what precisely is the rule for omitting a paper? Just that the paper itself cannot introduce a new method, or more stringently that none of the methods can have been developed by one of the authors?

2. There are notes on each paper in that list that suggest what criteria were used to rank methods and how the methods compared based on those criteria. But, it is not easy to understand what these notes mean, and it would be much better if the authors had explained this. I realize this would be a longer discussion, but it would improve interpretability of the results and (to some extent) enable reproducibility.

Also, I remain concerned about the limitation to 69 publications. The authors have responded by asserting that this is necessary (to avoid bias) and also to say that their power law figure justifies the restriction. But since there is no explanation for the figure (how the data are generated, how to interpret them, and why they are relevant), I don't know what to make of the figure. I do not find myself adequately convinced that this manuscript has enough data to draw strong conclusions about trends.

Related to this, in looking over this list of 69 papers, there seem to be quite a few about genome assembly and related tasks, multiple sequence alignment, and phylogeny estimation, and I can't help but wonder how representative this is of bioinformatics tools. Does this relatively low coverage of the bioinformatics tools help, or does it hurt? Some discussion would be helpful.

In conclusion, I still have concerns about the methodology (since it reduces data) and I do not find the supplementary materials adequate for this study in terms of interpretability. It might help for the authors to make sure that they either clarify exactly why it's okay for Julie Thompson to be an author on a paper studying ClustalW when she is an author (and the lead author!) on a paper about the method, or else remove that paper from the collection (and in that case also very carefully check the remaining papers).

But the paper is improved and the questions are interesting; whether the conclusions are that well supported is unclear to me, but this study may help stimulate other researchers to address the same or related questions, and that will be beneficial as well.

**Reviewer 2**

The authors' revised version has nearly completely addressed my original concerns. I am persuaded by the authors' argument that my remaining concerns are impractical to address within the scope of this manuscript and that they instead represent a distinct scholarly contribution.

**Reviewer 3**

The authors have adequately addressed all of my comments and concerns. I congratulate them on this manuscript that covers a very important (and not sufficiently well-studied) problem in our research area. While I would always be happy to see more discussion or interpretation of such data, I am also sympathetic of the authors' concerns about over-interpreting the data, and respect the level of speculation with which they are comfortable.

Note: There seem to be some issues with the journal-compiled version of the article (**all** of the citations were missing and replaced with "?"), I was able to find the modified article with marked changes included as a linked document in the submission. I will repeat this note to the editor as it seems an issue with the journal's compilation of the article and not the authors' original submission.

**Authors’ response**

In my earlier review, I asked specific questions that mainly had to do with understanding the

research protocol -- how the publications were screened, why the papers that were rejected

were rejected, which publications were included, what criteria were used to select the "best"

methods, etc. To some extent these are answered and the manuscript is now in better

shape. The low-level writing errors have been addressed. On the other hand, I still have

concerns the study design and how it is described in the paper and in the files provided in

their github repository. These are not

● This paragraph fragment appears to be incomplete. Is this a copy-paste error?

In particular, the authors point out a specific file (speed-vs-accuracytoolRanks2005-2020.tsv,

available in their github repository) which provides some insight into how they are

interpreting each paper. These interpretations are interesting and show substantial effort on

the part of the people doing the annotation - but they also reveal some problems.

1. The way the authors characterize the Boulesteix criteria for inclusion, then at least one

of the 69 publications that passed the acid test probably should not have passed (although

perhaps the authors should revise what their requirements are). Specifically, Thompson et

al., "A comprehensive benchmark study of multiple sequence alignments: Current

Challenges and Future Perspectives", is in the list, and this paper compares ClustalW to

many other alignment methods. But Julie Thompson is also the first author on the paper for

ClustalW. So what precisely is the rule for omitting a paper? Just that the paper itself cannot

introduce a new method, or more stringently that none of the methods can have been

developed by one of the authors?

● This highlights the difficulty of identifying conflicted authors, even ones as high-profile

as Dr Julie Thompson. We have removed the Thompson benchmark from our

analysis. Pleasingly, none of the results have significantly changed – thus

highlighting the result suggested by our power analysis, that we have more than

sufficient data to detect even modest effect sizes.

2. There are notes on each paper in that list that suggest what criteria were used to rank

methods and how the methods compared based on those criteria. But, it is not easy to

understand what these notes mean, and it would be much better if the authors had explained

this. I realize this would be a longer discussion, but it would improve interpretability of the

results and (to some extent) enable reproducibility.

● We have added additional comments to the github repository that add further details

for each field in the speed-vs-accuracytoolRanks2005-2020.tsv dataset.

Also, I remain concerned about the limitation to 69 publications. The authors have

responded by asserting that this is necessary (to avoid bias) and also to say that their power

law figure justifies the restriction. But since there is no explanation for the figure (how the

data are generated, how to interpret them, and why they are relevant), I don't know what to

make of the figure. I do not find myself adequately convinced that this manuscript has

enough data to draw strong conclusions about trends.

● We seem to have a mis-understanding here. We have not capped the analysis to 69

(or 68) benchmarks – this corresponds to 498 ranked methods which is really the unit

we are analysing here. At the time we gathered these benchmarks, they were the

bulk of the studies that we identified that met our inclusion criteria and were detected

with our literature mining method.

● Power analyses are a reasonably common statistical method to determine what is a

sufficient sample size to identify a given effect-size with a pre-specified significance

level.

Related to this, in looking over this list of 69 papers, there seem to be quite a few about

genome assembly and related tasks, multiple sequence alignment, and phylogeny

estimation, and I can't help but wonder how representative this is of bioinformatics tools.

Does this relatively low coverage of the bioinformatics tools help, or does it hurt? Some

discussion would be helpful.

● Alignment, assembly and phylogeny really is fundamental to a lot of bioinformatic

research. This is possibly why we were able to locate more benchmarks for these

areas. While delving into the potential similarities and differences between the

different sub-disciplines of bioinformatics would be very interesting, we think this is

outside the scope of the present study.

In conclusion, I still have concerns about the methodology (since it reduces data) and I do

not find the supplementary materials adequate for this study in terms of interpretability. It

might help for the authors to make sure that they either clarify exactly why it's okay for Julie

Thompson to be an author on a paper studying ClustalW when she is an author (and the

lead author!) on a paper about the method, or else remove that paper from the collection

(and in that case also very carefully check the remaining papers).

● This was a mistake on our part. Thanks to the reviewer for identifying it, the paper

has now been removed.

But the paper is improved and the questions are interesting; whether the conclusions are

that well supported is unclear to me, but this study may help stimulate other researchers to

address the same or related questions, and that will be beneficial as well.
